# Placental hormones, IGF-1, and early-life growth: endocrine links between birth size and infant metabolic programming—a systematic review

**DOI:** 10.1007/s00431-026-06985-5

**Published:** 2026-04-28

**Authors:** Ashraf T. Soliman, Fawzia Alyafei, Nada Alaaraj, Shayma Ahmed, Noor Hamed, Sohair Elsiddig

**Affiliations:** Department of Pediatrics, Hamad Medical Corporation, Doha, Qatar

**Keywords:** Placental hormones, IGF-1, Fetal growth, Small for gestational age, Preterm infant, Metabolic programming

## Abstract

The purpose of this study is to systematically review human evidence linking placental hormones and IGF-1 with fetal growth, birth size, and early-life metabolic programming, and to identify disease-specific translational signals relevant to fetal growth restriction, metabolically complicated pregnancy, and extreme prematurity. A systematic review of PubMed and Scopus was performed for human studies published from January 2000 to March 2025. Eligible studies examined placental growth hormone, human placental lactogen, placental IGF-axis markers, or related stress-hormone pathways in relation to birth size, fetal or infant growth, or early metabolic outcomes. Reporting was aligned with PRISMA 2020. Risk of bias was assessed using RoB 2 for randomized trials and ROBINS-I principles for observational studies. Because of substantial clinical and methodological heterogeneity, findings were synthesized narratively. Thirty-seven included studies showed that mid-gestation placental growth hormone had the most consistent positive associations with fetal growth and birth size, whereas early placental growth hormone was less informative. Cord blood IGF-1 was the most reproducible endocrine correlate of birth length and weight. Reduced maternal or placental lactogen signals and lower placental 11β-HSD2 activity clustered with placental insufficiency and fetal growth restriction, while diabetic pregnancies showed a shift toward adiposity-related outcomes. In extreme prematurity, abrupt loss of placental endocrine support created a translational window in which rhIGF-1/rhIGFBP-3 replacement emerged as the clearest mechanism-based intervention, although current clinical evidence remains preliminary.

*Conclusion*: Placental endocrine markers should not be used as standalone screening tools. Their main value at present is mechanistic and translational: combined hormone patterns may refine biological understanding and risk stratification within defined maternal and neonatal disease frameworks, while IGF-1 replacement in extreme prematurity represents the most credible current therapeutic lead.

**Table:** 

**What is Known:** • *Placental hormones and IGF-1 are linked with fetal growth, birth size, and neonatal metabolic adaptation, but 45 published evidence is heterogeneous and clinically fragmented.* **What is New:** • *This systematic review organizes the human data within fetal growth restriction, diabetic/LGA pregnancy, and extreme prematurity frameworks and identifies translational signals—especially early IGF-1 replacement in extreme prematurity—rather than standalone diagnostic biomarkers.*

**Graphical Abstract:**

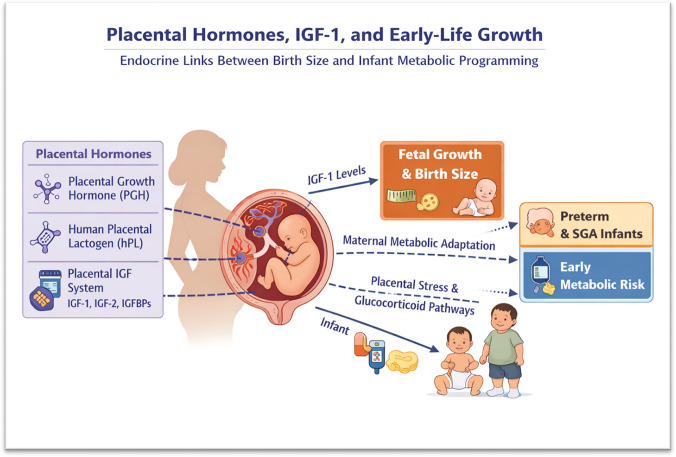

**Supplementary Information:**

The online version contains supplementary material available at 10.1007/s00431-026-06985-5.

## Introduction

The placenta is a transient but highly active endocrine organ that plays a pivotal role in regulating fetal growth, neonatal adaptation, and early-life metabolic programming. Beyond its transport function, the placenta secretes multiple hormones that coordinate maternal physiology with fetal growth demands, thereby influencing birth size, proportionality, and postnatal growth trajectories [1, 2]. Increasing evidence indicates that disturbances in placental endocrine function contribute to fetal growth restriction (FGR), prematurity-related growth failure, and long-term metabolic risk in childhood [3, 4].

Fetal linear growth differs fundamentally from postnatal growth regulation. During intrauterine life, growth hormone (GH) is not the primary driver of growth; instead, fetal growth is largely dependent on insulin, insulin-like growth factors (IGFs), and placental endocrine signals that modulate nutrient availability and anabolic signaling [5, 6]. This distinction is clinically relevant, as infants with congenital GH deficiency are typically born with normal length, whereas disorders affecting IGF signaling or placental function are associated with significant intrauterine growth impairment [7, 8]. These observations highlight the central role of placental–IGF interactions in determining early growth outcomes.

Several placental hormones, including placental growth hormone, human placental lactogen, and components of the placental IGF system, have been associated with fetal growth patterns in human studies. Alterations in these hormones have been reported in pregnancies complicated by placental insufficiency, maternal metabolic disease, or preterm delivery [9–12]. However, the relative contribution of individual hormones, their interrelationships, and their relevance to postnatal linear growth and metabolism remain incompletely defined, particularly from a pediatric perspective.

Cord blood IGF-1 has emerged as a reproducible biomarker linking placental endocrine function to fetal size and early body composition [13, 14]. Low IGF-1 concentrations at birth are consistently observed in infants born small for gestational age or preterm and are associated with impaired postnatal growth and altered metabolic trajectories [15, 16]. These findings suggest that placental endocrine signals exert effects that extend beyond birth and may influence growth and metabolic regulation throughout infancy.

Preterm birth represents a unique clinical model in which placental endocrine support is abruptly interrupted during a critical developmental window. Extreme preterm infants exhibit markedly reduced circulating IGF-1 levels, poor linear growth, and disproportionate fat accretion despite optimized nutritional support [17, 18]. This constellation of findings has been conceptualized as a state of early-life endocrine and metabolic disruption, underscoring the importance of placental hormones in shaping postnatal physiology [19].

Despite substantial progress over the past two decades, the literature on placental hormones remains fragmented across obstetric, neonatal, and pediatric endocrinology disciplines. To improve clinical and translational relevance, this systematic review interprets the evidence within predefined maternal and neonatal disease frameworks—placental insufficiency/fetal growth restriction, metabolically complicated pregnancy, and extreme prematurity to identify biologically meaningful patterns, hypothesis-generating markers, and potential therapeutic leverage points.

## Objectives

- **To synthesize evidence from the past 25 years on the role of major placental hormones**, including placental growth hormone, human placental lactogen, and the placental IGF system—in regulating fetal and early postnatal linear growth.
- **To examine the relationship between placental endocrine function and IGF-1 concentrations** in cord blood and early infancy, and their associations with birth size, growth trajectories, and body composition.
- **To evaluate the implications of placental hormone disruption for neonatal and infant metabolism**, particularly in preterm and growth-restricted infants, with relevance to pediatric diagnosis, management, and long-term prognosis.

## Methods

### Study design

This study was conducted as a systematic review with narrative synthesis and was reported in accordance with the PRISMA 2020 statement. The review focused on human studies examining placental endocrine pathways and their associations with fetal growth, birth size, early postnatal growth, or neonatal metabolic programming. Meta-analysis was not planned because marked heterogeneity was expected in populations, assay methods, sampling windows, and outcome definitions.

### Data sources and search strategy

A comprehensive search of PubMed and Scopus was performed for articles published between January 2000 and March 2025. Search terms combined placental growth hormone, placental lactogen, insulin-like growth factor, IGF-binding protein, placenta, cord blood, fetal growth restriction, preterm infant, and birth size. Titles and abstracts were screened for relevance, followed by full-text review when eligibility was uncertain. Reference lists of key articles were also reviewed to identify additional human studies.

### Eligibility criteria

Included studies met the following criteria:

- Human studies (cohort, case–control, cross-sectional, or randomized trials)
- Measurement of placental hormones or related endocrine markers (PGH, hPL, IGF-1, IGF-2, IGF-binding proteins)
- Reported outcomes related to fetal growth, birth length/weight, postnatal linear growth, or metabolic indicators in neonates or infants

Studies were excluded if they lacked growth or metabolic outcomes, were limited to animal models without human correlation, or consisted solely of case reports or expert opinion.

### Data extraction and synthesis

Data were extracted on study design, population characteristics, gestational age, hormonal measurements, comparator groups, growth outcomes, and metabolic endpoints. Findings were synthesized narratively and organized into clinically relevant disease frameworks: placental insufficiency/fetal growth restriction, metabolically complicated pregnancy, and extreme prematurity.

### Quality assessment

Study quality and risk of bias were appraised using Cochrane-based approaches. Randomized controlled trials were evaluated with RoB 2, while cohort, case–control, and cross-sectional studies were judged according to ROBINS-I principles, with particular attention to confounding by maternal phenotype, timing of sampling, placental heterogeneity, and neonatal illness severity.

### Statistical considerations

No new pooled statistical analyses were performed. Reported associations, adjusted models, and effect sizes from the original studies were interpreted qualitatively, with emphasis on consistency, temporality, biological plausibility, and whether findings suggested a potentially modifiable pathway or a therapeutic development signal.

## Results

**Fig. 1:**
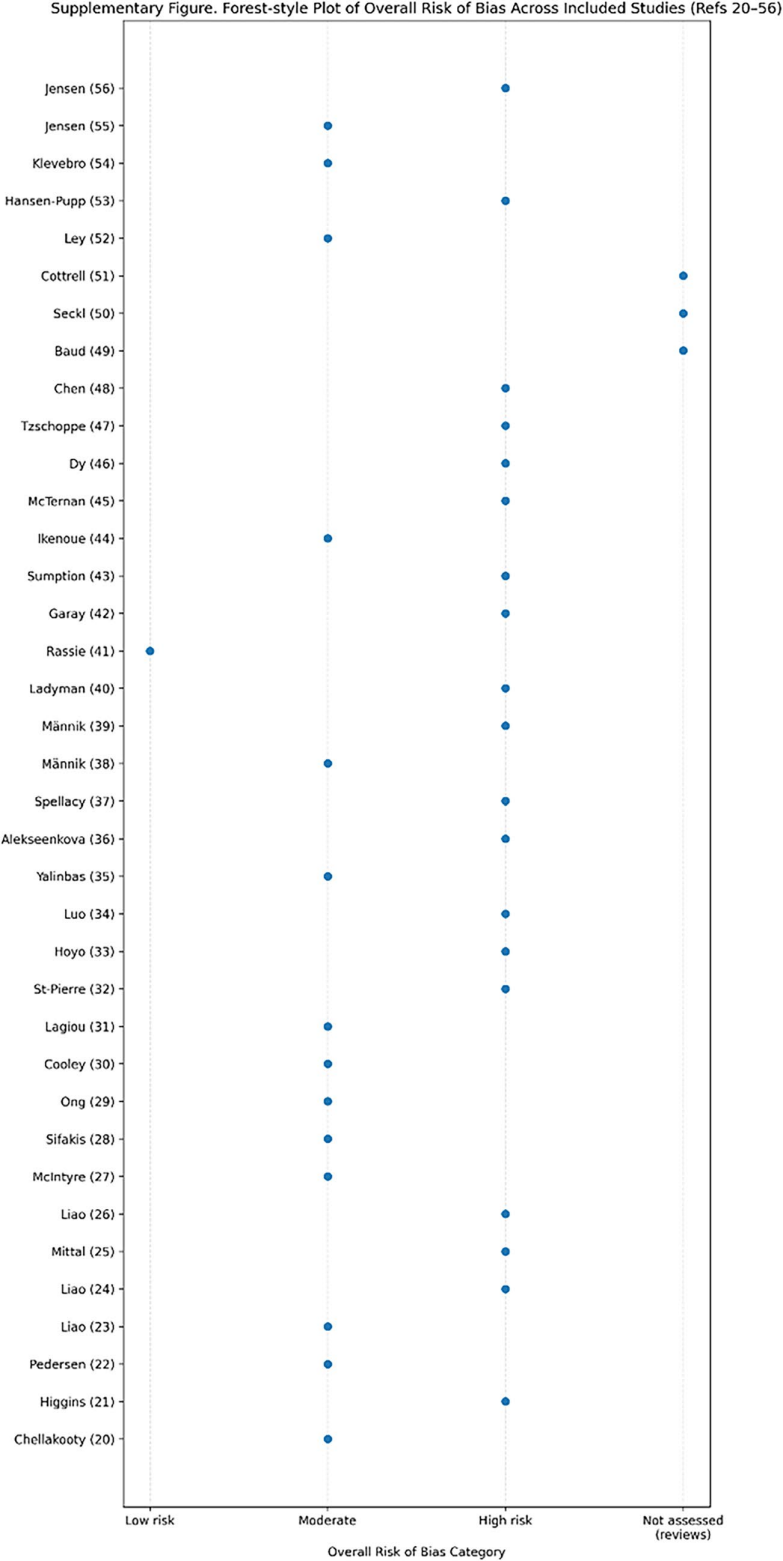
Placental hormones as central regulators of fetal growth and early-life metabolic programming. This graphical abstract illustrates the **central coordinating role of placental hormones** in linking maternal physiology to fetal growth and early-life metabolic programming. It clearly conveys how placental growth hormone, human placental lactogen, and the placental IGF system act through **both direct endocrine pathways (IGF-1–mediated fetal growth)** and **indirect mechanisms (maternal metabolic adaptation and glucocorticoid regulation)** to influence birth size, proportionality, and postnatal growth trajectories. The downstream emphasis on **preterm and SGA infants** appropriately highlights populations most vulnerable to disrupted placental signaling and subsequent metabolic risk

The key human evidence is summarized in Tables 1, 2, 3, and 4. Placental stress hormone and glucocorticoid-barrier findings are provided in Supplementary Table S1, the overall risk-of-bias synthesis in Supplementary Table S2, and the overall risk-of-bias plot in Supplementary Figure S1. Figure 1 presents the integrative biological model linking placental endocrine function with fetal growth and early metabolic programming.

**Table 1:** Placental growth hormone (PGH; GH-V) and fetal growth/birth size associations (human studies)

Study	Population/design	Hormone measure	Growth outcomes	Main finding
Chellakooty et al., JCEM 2004 [20]	Prospective longitudinal (uncomplicated pregnancies)	Maternal PGH, IGF-I, IGFBP-3 (serial)	Serial ultrasound growth; birth size	Maternal PGH and IGF-axis markers closely tracked fetal growth trajectories and birth size
Higgins et al., PLoS One 2012[11]	Case–control (type 1 diabetes vs controls)	Maternal and fetal PGH; maternal IGF-I; IGFBP-3	Fetal skeletal indices; birth-weight centile	Maternal PGH correlated with fetal growth and birth-weight centile; fetal PGH detectable but showed weaker associations
Pedersen et al., Ultrasound Obstet Gynecol 2010[21]	Prospective cohort (early mid-gestation)	Maternal PGH, hPL, IGF-1	Mid-gestation fetal biometry; birth size	Maternal PGH, but not hPL or IGF-1, was positively associated with fetal growth in the first half of pregnancy
Liao et al., Growth Factors 2016[22]	Nested case–control (AGA vs SGA vs LGA)	Maternal GH-V at ~ 20 weeks	Birth weight; customized birth-weight centile	GH-V concentrations were higher in LGA pregnancies and positively correlated with birth-weight centiles
Liao et al., Hormones (Athens) 2017[23]	Nested case–control (GDM vs controls)	Maternal GH-V, IGF-1, IGF-2, IGFBP-1/3	LGA/AGA outcomes; birth size	GH-V is not globally elevated in GDM, but higher in GDM pregnancies delivering LGA infants
Mittal et al., J Matern Fetal Neonatal Med 2007[24]	Case–control (preeclampsia)	Maternal and fetal PGH	Birth weight; growth restriction context	PGH increased in maternal and fetal serum in preeclampsia, reflecting altered placental endocrine function
Liao et al., Pregnancy Hypertens 2017[25]	Nested case–control (preeclampsia vs controls)	Maternal GH-V, IGF-1, IGFBP-1/3 at ~ 20 weeks	Birth size; PE-related outcomes	Mid-pregnancy GH-V and IGF-axis markers differed in pregnancies complicated by preeclampsia
McIntyre et al., Horm Metab Res 2002[26]	Physiological study (normal pregnancy)	Maternal PGH during OGTT	Birth weight correlation	PGH not suppressed by glucose loading; maternal metabolic milieu influenced birth-weight associations
Sifakis et al., Ultrasound Obstet Gynecol 2012[27]	First-trimester screening cohort	Maternal PGH at 11–13 weeks (MoM)	Birth-weight percentile; SGA	First-trimester PGH showed no meaningful association with SGA or birth-weight percentile

**Table 2:** Placental IGF-1/IGF-2 (maternal, cord blood, and placental epigenetic IGF-2 markers) and fetal growth/birth size (human studies)

Study	Population/design	IGF measure (matrix/timing)	Growth outcomes	Main finding
Ong et al., JCEM 2000[28]	Term singleton cohort (*n*≈199)	Cord blood IGF-I, IGF-II, soluble IGF-2R, insulin, IGFBP-1/3	Birth weight, length, head circumference, ponderal index, placental weight	Cord IGF-I and insulin positively related to multiple size-at-birth measures; IGF-II and IGF-2R related to birth size/placental metrics, supporting IGF-II’s role in fetal/placental growth
Cooley et al., Eur J Obstet Gynecol Reprod Biol 2004[29]	Maternal cord paired sampling study	Maternal antenatal serum IGF-1/IGF-2/IGFBP-3 + cord blood IGF-1/IGF-2/IGFBP-3	Birth weight/length correlations (reported)	Demonstrated maternal–fetal coupling of IGF axis at delivery; provides paired maternal/cord context for growth associations
Mexitalia et al., Journal of Clinical Neonatology 2021(30)	Cohort study	Cord blood IGF-1 and leptin	Birth weight	Birth weight was correlated with the level of IGF-1 and leptin, and maternal body mass index was correlated with cord blood leptin
St-Pierre et al., Epigenetics 2012[31]	Human birth cohort	Placental IGF-2 DNA methylation (DMR0/DMR2) + maternal IGF-2	Birth weight/fetal growth indices	Placental IGF-2 methylation patterns correlated with fetal growth indices, supporting regulation of IGF-2 as a growth modulator
Hoyo et al., Cancer Causes Control 2012[32]	Birth cohort	Cord blood IGF-2 + cord methylation fractions at imprinted loci	Birth weight	Higher IGF-2 concentrations are associated with higher birth weight after adjustment; links IGF-2 biology with size at birth
Luo et al., JCEM 2012[33]	Maternal–fetal study incl. GDM	Maternal mid/late gestation IGF-I/IGF-II + fetal/cord IGF-I/IGF-II	Birth weight (hypertrophy/LGA context)	Higher maternal IGF-I (more than IGF-II) associated with greater fetal/placental growth; IGF-I implicated in fetal hypertrophy in GDM
Yalinbas et al., Am J Perinatol 2019[34]	Term infants categorized SGA/AGA/LGA	Cord blood IGF-I and IGF-II (plus adipokines)	SGA vs AGA vs LGA; birth size indices	Cord IGF-I/IGF-II differed by growth category, supporting lower IGF levels in growth-restricted groups (with concurrent adipokine patterning)
Alekseenkova et al., Int J Gynaecol Obstet 2023[34]	Pregnancy cohort (first-trimester biomarkers)	Maternal first-trimester IGF-1 and IGF-1/IGFBP-1	Macrosomia/large size at birth	First-trimester maternal IGF-1–related thresholds showed discriminatory value for later macrosomia risk (growth excess phenotype)

**Table 3:** Placental lactogens (human placental lactogen; hPL/CSH) and growth restriction signals (human studies)

Study	Population/design	Measure	Outcome	Main finding
Spellacy et al., 1976 [35]	Normal vs IUGR pregnancies (late gestation sampling)	Maternal serum hPL after 36 weeks	Fetal + placental weight	Maternal hPL values were lower in IUGR across late gestation, consistent with reduced placental endocrine function
Männik et al., 2010 [36]	Term placentas across outcomes (incl. SGA/LGA strata)	Placental CSH1/CSH2 (hPL genes) and GH/CSH cluster expression	Birth weight/size at birth	Placental GH/CSH gene-cluster expression (including CSH1/2) associated with birth weight, supporting placental lactogen axis relevance to fetal growth
Männik et al., 2012 [37]	Case–control placental transcript study (preeclampsia ± growth effects; GD; LGA)	Placental CSH1-related transcripts (incl. alternative transcripts)	Growth-related phenotypes within PE/LGA contexts	Identified reduced CSH1-related transcript expression in PE subgroups, supporting placental lactogen-pathway disruption in placental disease states linked to growth impairment
Ladyman et al., 2021 [38]	Mid-pregnancy case–control (SGA vs controls; SCOPE cohort)	Maternal plasma placental lactogen (mid-gestation) ± prolactin	SGA birth outcome	Tested whether mid-gestation placental lactogen differs in pregnancies later delivering SGA; provides contemporary human evidence on SGA prediction/association
Rassie et al., 2022 [39]	Systematic review + meta-analysis	Maternal circulating hPL in relation to metabolic conditions and fetal growth	Birth weight/placental mass; metabolic outcomes	Synthesized observational evidence showing hPL relates to maternal metabolic state, and in diabetes-affected pregnancies hPL tends to be positively related to placental mass and infant birth weight
Garay et al., 2022 [40]	Pregnancy cohort (behavior/metabolic predictors; adjusted models)	Maternal hPL (with placental weight adjustment)	Customized birth-weight centile; LGA/size outcomes	Maternal hPL showed positive associations with customized birth-weight centiles in adjusted analyses, supporting hPL as a marker of the endocrine capacity underpinning growth variation
Sumption et al., 2020 [41]	Term cohort (maternal serum, psychosocial outcomes)	Maternal serum hPL at term	Birth size context + postpartum outcomes	Reports term hPL distribution in contemporary pregnancy; paper cites prior evidence that lower term hPL associates with SGA/FGR phenotypes in related cohorts

**Table 4:** IGF-1/IGFBP-3 replacement or IGF-axis–guided interventions in preterm/SGA: growth and metabolic endpoints (human studies)

Study	Population/design	Intervention/exposure	Growth and metabolic endpoints	Main finding
Ley et al., J Pediatr 2019[50]	Extremely preterm infants (GA 23–27 weeks); multicenter phase 2 RCT	Continuous IV rhIGF-1/rhIGFBP-3 vs standard care	Primary: ROP severity; Key secondary: severe BPD, severe IVH; safety	rhIGF-1/rhIGFBP-3 did not reduce ROP, but was associated with lower severe BPD and a nonsignificant reduction in severe IVH; safety reported
Hansen-Pupp et al., Growth Horm IGF Res 2017[51]	Extreme preterm infants; phase II feasibility analysis (within the same program)	Continuous IV rhIGF-1/rhIGFBP-3 titrated to fetal-physiologic IGF-1 targets	Achievement of target IGF-1 levels, tolerability; neonatal morbidity signals	Continuous infusion raised serum IGF-1 toward target fetal ranges and was feasible/well tolerated; supports practical delivery of replacement strategy
Klevebro et al., Growth Horm IGF Res 2020[52]	Extremely preterm infants receiving rhIGF-1/rhIGFBP-3 (trial cohort analysis)	Biomarker analysis during replacement infusion	IGF-1 attainment, inflammation-IGF interaction (IL-6), IGFBP-1	Higher IL-6 and IGFBP-1 predicted lower achieved IGF-1 during infusion—suggesting inflammation can blunt replacement efficacy
Jensen et al., Eur J Endocrinol 2014[53]	Short children born SGA; multicenter randomized trial (NESGAS)	IGF-1–titrated GH dosing vs conventional GH dosing	Linear growth response; IGF-1 levels; safety/metabolic monitoring	IGF-1 titration achieved more “physiologic” IGF-1 exposure but produced less growth response than standard dosing, supporting heterogeneity/relative IGF resistance in short SGA children
Jensen et al., Horm Res Paediatr 2013[54]	SGA children starting GH (multicenter prospective study; NESGAS)	GH therapy with baseline IGF-I stratification	Insulin secretion and insulin sensitivity over first year; IGF-I predictors	Baseline IGF-I helped predict insulin secretion/sensitivity changes during GH therapy, linking IGF-axis status to metabolic response in SGA management

Placental growth hormone (PGH; GH-V) associations with fetal growth and birth size are summarized in Table 1.

Across validated human studies, **maternal PGH measured in mid-gestation** shows the most consistent association with **fetal growth and birth size**, whereas **first-trimester PGH** has limited predictive value for SGA or growth outcomes.

Key human studies on placental IGF-1/IGF-2 markers (maternal, cord blood, and placental epigenetic IGF-2 indicators) and birth size are summarized in Table 2.

Across validated human cohorts, **cord blood IGF-1** and **IGF-2** generally correlate positively with **birth size (including length)**, while **maternal IGF-1** (more than IGF-2) shows more consistent links to fetal/placental growth in pregnancy complication contexts (notably GDM). Placental **IGF-2 epigenetic regulation** provides additional human evidence connecting IGF-2 biology with fetal growth variation.

Human evidence on placental lactogens (hPL/CSH) and growth restriction phenotypes is summarized in Table 3.

Across human studies, **lower maternal circulating hPL** and/or **reduced placental CSH (hPL gene) expression** are recurrent signals in **placental dysfunction states** and are frequently linked to **lower birth size/SGA/FGR-related phenotypes**, while systematic synthesis also supports hPL as an indicator of placental endocrine activity tightly coupled to maternal metabolic adaptation.

Human studies of placental stress hormones and glucocorticoid-barrier pathways relevant to growth and programming are summarized in Supplementary Table S1.

Interventional and IGF-axis–guided studies in preterm or SGA populations are summarized in Table 4.

In extremely preterm infants, **physiological IGF-1 replacement (rhIGF-1/rhIGFBP-3)** is feasible and improves attainment of fetal-range IGF-1, with trial data showing **no ROP benefit** but a signal toward **reduced severe BPD**; inflammatory activity may impair target attainment. In SGA children, IGF-1–guided GH strategies highlight **variable IGF sensitivity** and demonstrate that “normalizing IGF-1” does not necessarily maximize linear growth, while metabolic responses to GH depend partly on baseline IGF status.

Placental stress hormones and glucocorticoid-barrier pathways: growth and programming links (human evidence) are summarized in Supplementary Table S1.

Human studies consistently link placental CRH and reduced placental 11β-HSD2 activity with placental insufficiency, impaired fetal growth, and altered early metabolic programming risk.

The overall risk-of-bias synthesis across included studies is summarized in Supplementary Table S2.

Most included studies were observational and therefore carried moderate to high risk of bias, mainly from confounding, selection effects, placental heterogeneity, and clinical complexity.

A graphical overview of the placental endocrine network discussed in this review is provided in Fig. 1.

The overall risk of bias across included studies is summarized in Supplementary Figure S1.

This Forest-style summary of overall risk of bias for studies included in the review [11, 20–54]. Each point represents an individual study, classified using Cochrane RoB 2 or ROBINS-I principles.

## Discussion

This review integrates human evidence from the past 25 years to clarify how placental hormones are shared in the regulation of fetal linear growth, IGF-1 availability, and early metabolic programming [11, 20–25].

Accumulating evidence supports a central role for placental growth hormone (PGH) in shaping fetal growth trajectories, primarily through effects on maternal metabolism and the IGF axis. Longitudinal studies demonstrate that rising maternal PGH concentrations correlate with maternal IGF-1 levels and fetal growth velocity, particularly in mid-gestation [20, 21]. These associations are weaker or absent in early pregnancy, limiting the predictive value of first-trimester PGH for growth restriction [27]. Collectively, the evidence supports a model in which PGH enhances maternal nutrient availability via increased insulin resistance and lipolysis, thereby indirectly supporting fetal growth [20, 26].

In metabolically complicated pregnancies, including type 1 diabetes and gestational diabetes, PGH associations shift toward fetal adiposity and large-for-gestational-age phenotypes rather than proportional linear growth [11, 23]. This context dependency points to the importance of maternal metabolic status and placental efficiency in modulating PGH effects.

The placental IGF axis emerges as a key determinant of fetal size and early postnatal growth. Cord blood IGF-1 consistently correlates with birth length and weight, reflecting integrated placental nutrient sensing and fetal anabolic signaling [28–30]. In contrast, IGF-2 appears more closely linked to placental growth and transport capacity, acting predominantly at the placental level [31, 32].

Epigenetic alterations in IGF-related genes reported in growth-restricted or metabolically stressed pregnancies provide a plausible mechanistic link between the intrauterine environment and long-term growth and metabolic outcomes [31, 32]. These data support the concept of the IGF system as both a growth effector and a mediator of developmental programming.

Human placental lactogen (hPL) reflects placental mass and endocrine function rather than acting as a direct fetal growth hormone. Lower maternal hPL concentrations and reduced placental chorionic somatomammotropin gene expression are repeatedly observed in growth-restricted and placental dysfunction states [35–37]. Systematic synthesis further indicates that hPL plays a key role in maternal metabolic adaptation, particularly insulin resistance, thereby indirectly influencing fetal nutrient supply [39].

Despite consistent associations, hPL lacks specificity for linear growth versus adiposity, supporting its interpretation as an integrative marker of placental endocrine capacity rather than a standalone predictor.

Placental stress hormones are critical modulators of growth and metabolic programming. Elevated placental corticotropin-releasing hormone (CRH) is associated with altered fetal blood-flow distribution favoring hepatic perfusion, potentially prioritizing short-term metabolic adaptation over linear growth [42]. In parallel, reduced placental 11β-hydroxysteroid dehydrogenase type 2 (11β-HSD2) expression or activity increases fetal glucocorticoid exposure and is consistently linked to growth restriction and altered postnatal growth trajectories [43–45]. Emerging cohort data further associate placental 11β-HSD2 expression with early insulin sensitivity in infancy, extending the relevance of placental stress regulation beyond birth size to metabolic programming [46]. These findings align with established developmental origins frameworks [47–49].

Interventional studies remain limited but informative. In extremely preterm infants, continuous replacement with recombinant IGF-1/rhIGFBP-3 restores circulating IGF-1 toward physiological fetal levels and provides the clearest current example of a mechanism-based therapeutic strategy arising from placental endocrinology. The negative primary ROP endpoint requires caution, but biologic signals in bronchopulmonary dysplasia and intraventricular hemorrhage support continued evaluation in phenotype-enriched trials.

When the evidence is organized by disease framework, distinct endocrine patterns emerge. In placental insufficiency and fetal growth restriction, lower mid to late gestation PGH and hPL signals, together with reduced placental 11β-HSD2 activity, are best interpreted as markers of impaired placental function, altered nutrient partitioning, and excessive fetal glucocorticoid exposure. In contrast, diabetic pregnancies shift the endocrine phenotype toward enhanced fetal adiposity and large-for-gestational-age outcomes, indicating that the biologic meaning of a hormone signal depends on maternal metabolic context rather than on a single universal threshold.

These observations have translational value because they point to modifiable pathways rather than to standalone diagnostic tests. Combined endocrine patterns may help refine risk stratification or enrich future intervention trials, but they should be interpreted alongside fetal biometry, Doppler studies, placental phenotype, and neonatal clinical status. Future studies should therefore move from descriptive hormone-outcome associations toward disease-specific models that test causality, define therapeutic windows, and determine whether endocrine-guided stratification can improve clinically meaningful outcomes.

Collectively, the evidence supports a network model of placental endocrine regulation, in which PGH, IGFs, lactogens, and stress hormones interact to balance fetal growth demands with maternal metabolic capacity. Disruption at any node—placental mass, IGF signaling, or glucocorticoid-barrier function—can shift growth trajectories toward restriction or disproportionate adiposity [11, 20–54]. The marked heterogeneity across studies reflects sensitivity to maternal phenotype, placental health, and gestational timing.

Clinically, these findings indicate that no single placental hormone reliably predicts growth outcome. Rather, combined hormonal patterns integrated with fetal biometry and Doppler indices may better identify fetuses at risk for adverse growth and metabolic programming. While routine placental hormone measurement is not currently recommended, understanding these pathways informs postnatal surveillance and risk stratification of infants born preterm or small for gestational age.

## Conclusion

This review demonstrates that placental hormones play a central and coordinated role in regulating fetal linear growth, IGF-1 availability, and early-life metabolic programming. Human evidence consistently supports a network model in which placental growth hormone, placental lactogens, the IGF system, and glucocorticoid-regulating pathways interact to balance fetal growth demands with maternal metabolic capacity. Disruption of these pathways, particularly in preterm birth and fetal growth restrictions, is associated with impaired postnatal growth trajectories and early metabolic vulnerability. Although heterogeneity and reliance on observational data limit causal inference, the convergence of endocrine, epigenetic, and interventional findings underscores the clinical relevance of placental hormone biology. Integrating placental endocrine insights into pediatric growth assessment may improve early risk stratification, guide follow-up, and inform future targeted interventions aimed at optimizing growth and metabolic health across the life course.

## Recommendations

- **Routine measurement** **of placental hormones is not recommended** in standard clinical practice, as current evidence does not support their use as standalone diagnostic or screening tools.
- **Selective measurements (research or high-risk settings)**—such as placental growth hormone, hPL, or cord blood IGF-1—may be considered in preterm, growth-restricted, or metabolically complicated pregnancies to improve pathophysiological understanding and risk stratification.
- **Clinical focus should remain on integrated assessment**, combining fetal biometry, Doppler studies, birth anthropometrics, and postnatal growth patterns, with placental hormone data used as supportive or exploratory biomarkers rather than primary decision drivers.

## Strengths

This review provides a comprehensive, pediatric-focused synthesis of human evidence over the past 25 years, integrating placental endocrinology with fetal linear growth, IGF-1 regulation, and early metabolic programming. A major strength is the structured integration of observational cohorts, mechanistic placental studies, and interventional trials, allowing biologically plausible pathways to be linked with clinically relevant growth and metabolic outcomes. The use of validated PubMed- and Scopus-indexed references, serial citation, and a formal Cochrane-style risk-of-bias assessment enhances methodological rigor and transparency. In addition, the emphasis on preterm and growth-restricted infants strengthens clinical relevance for pediatric endocrinologists and neonatologists.

## Limitations

The review is limited by the predominance of observational studies with moderate to high risk of bias, restricting causal inference. Considerable heterogeneity in study design, hormone assays, gestational timing, and outcome definitions precluded quantitative meta-analysis and necessitated narrative synthesis. Interventional evidence targeting placental or IGF-axis pathways remains sparse and confined to selected populations, limiting generalizability. Finally, long-term follow-up data linking placental hormone profiles to childhood and adolescent metabolic outcomes are limited, highlighting the need for well-designed longitudinal and interventional studies.

## Supplementary Information

Below is the link to the electronic supplementary material.

ESM 1 (DOCS 104 KB)

## Data Availability

No datasets were generated or analysed during the current study.

## Abbreviations

11β-HSD2: 11β-Hydroxysteroid dehydrogenase type 2
BPD: Bronchopulmonary dysplasia
CRH: Corticotropin-releasing hormone
FGR: Fetal growth restriction
hPL: Human placental lactogen
IGF: Insulin-like growth factor
IGFBP: IGF-binding protein
IUGR: Intrauterine growth restriction
PGH: Placental growth hormone
PRISMA: Preferred Reporting Items for Systematic Reviews and Meta-Analyses
ROP: Retinopathy of prematurity
SGA: Small for gestational age
