# Supplementary material for: Placental hormones, IGF-1, and early-life growth: endocrine links between birth size and infant metabolic programming—a systematic review
Source: Eur J Pediatr. 2026 Apr 28;185(5):313. doi: 10.1007/s00431-026-06985-5 (PMC13124825; doi:10.1007/s00431-026-06985-5)
Supplement: Supplementary file 1 — (DOCS 104 KB) [file 431_2026_6985_MOESM1_ESM.docx]

**Supplementary Material**

Placental Hormones, IGF-1, and Early-Life Growth: Endocrine Links Between Birth Size and Infant Metabolic Programming—A Systematic Review

.

**Supplementary Table S1. Placental stress hormones and glucocorticoid-barrier pathways: growth and programming links (human evidence)**

| **Study** | **Population / Design** | **Placental factor (measure)** | **Outcome** | **Main finding** |
| --- | --- | --- | --- | --- |
| Ikenoue et al., JCEM 2021 [44] | Prospective cohort (uncomplicated pregnancies; late gestation) | Placental CRH (maternal circulating; ~third trimester) | Fetal liver blood flow (Doppler) ± growth/body composition indices | Higher placental CRH was positively associated with fetal liver blood flow, supporting a substrate-partitioning signal relevant to fetal growth and body composition. |
| McTernan et al., J Clin Endocrinol Metab 2001 [45] | Placental tissue study: normal gestation vs IUGR | Placental 11β-HSD2 mRNA expression | IUGR status; inferred fetal GC exposure | Placental 11β-HSD2 expression was reduced in IUGR pregnancies, consistent with impaired glucocorticoid “barrier” in growth-restricted states. |
| Dy et al., Placenta 2008 [46] | Case–control (idiopathic IUGR vs controls) | Placental 11β-HSD2 activity + mRNA; umbilical cortisol/cortisone ratio | Birth size / IUGR phenotype | Demonstrated reduced placental 11β-HSD2 activity/mRNA in IUGR and a lower cortisone:cortisol ratio in umbilical artery, linking barrier impairment with fetal glucocorticoid exposure. |
| Tzschoppe et al., Pediatr Res 2009 [47] | AGA vs IUGR infants with placental gene expression | Placental 11β-HSD2 gene expression at birth | Birth length; infant growth velocity (first year) | Higher placental 11β-HSD2 expression correlated with greater birth length and related to postnatal growth dynamics in IUGR, supporting programming relevance. |
| Chen et al., Diabetes Care 2019 [48] | Prospective birth cohort | Placental 11β-HSD2 expression | Infant insulin resistance indicators | Higher placental 11β-HSD2 expression was associated with lower insulin resistance in infancy, linking placental GC-barrier function with early metabolic programming. |
| Baud et al., Front Endocrinol 2019 [49] | Narrative review (human + translational) focused on IUGR | Glucocorticoids; placental 11β-HSD2 and endocrine milieu | Growth restriction consequences; neurodevelopmental/metabolic vulnerability | Synthesizes evidence that glucocorticoid-related pathways and placental endocrine dysregulation contribute to IUGR sequelae and later vulnerability. |
| Seckl et al., Nat Clin Pract Endocrinol Metab 2007 [50] | Mechanisms review with human evidence | Glucocorticoids; placental metabolism (11β-HSD2) | Birth weight; later-life cardiometabolic/HPA outcomes | Integrates human and experimental evidence supporting placental 11β-HSD2 as a key regulator of fetal GC exposure; summarizes links to low birth weight and programmed disease risk. |
| Cottrell et al., Acta Physiol 2014 [51] | Developmental programming review (human + translational) | Fetoplacental 11β-HSD2 | Fetal growth; programming outcomes | Reviews evidence positioning fetoplacental 11β-HSD2 as a “hub” influencing fetal growth and long-term programming (includes human relevance). |

Summary: Human studies consistently link placental CRH and reduced placental 11β-HSD2 activity with placental insufficiency, impaired fetal growth, and altered early metabolic programming risk.

**Supplementary Table S2. Overall risk-of-bias assessment across included studies (Cochrane-style synthesis)**

| **Risk category** | **Risk assessment (Cochrane interpretation)** | **Reference numbers*** | **Number of studies** |
| --- | --- | --- | --- |
| Low risk of bias | Low risk across all key domains; results unlikely to be affected by bias | [41] | 1 |
| Moderate risk of bias | Some concerns in ≥1 domain (mainly confounding or attrition); results likely valid but should be interpreted with caution | [20], [22], [23], [27], [28], [29], [30], [31], [35], [38], [44], [52], [54] | 13 |
| High risk of bias | Serious concerns in ≥1 critical domain (confounding, selection bias, exposure misclassification); results vulnerable to bias | [21], [24], [25], [26], [32], [33], [34], [36], [37], [39], [40], [42], [45], [46], [47], [48], [53], [55], [56] | 19 |
| Narrative / mechanistic reviews† | Not formally assessed by RoB2 or ROBINS-I; credibility judged qualitatively | [49], [50], [51] | 3 |

Summary: Most included studies were observational and therefore carried moderate to high risk of bias, mainly from confounding, selection effects, placental heterogeneity, and clinical complexity.

**Supplementary Figure S1. Overall risk of bias across included studies.**


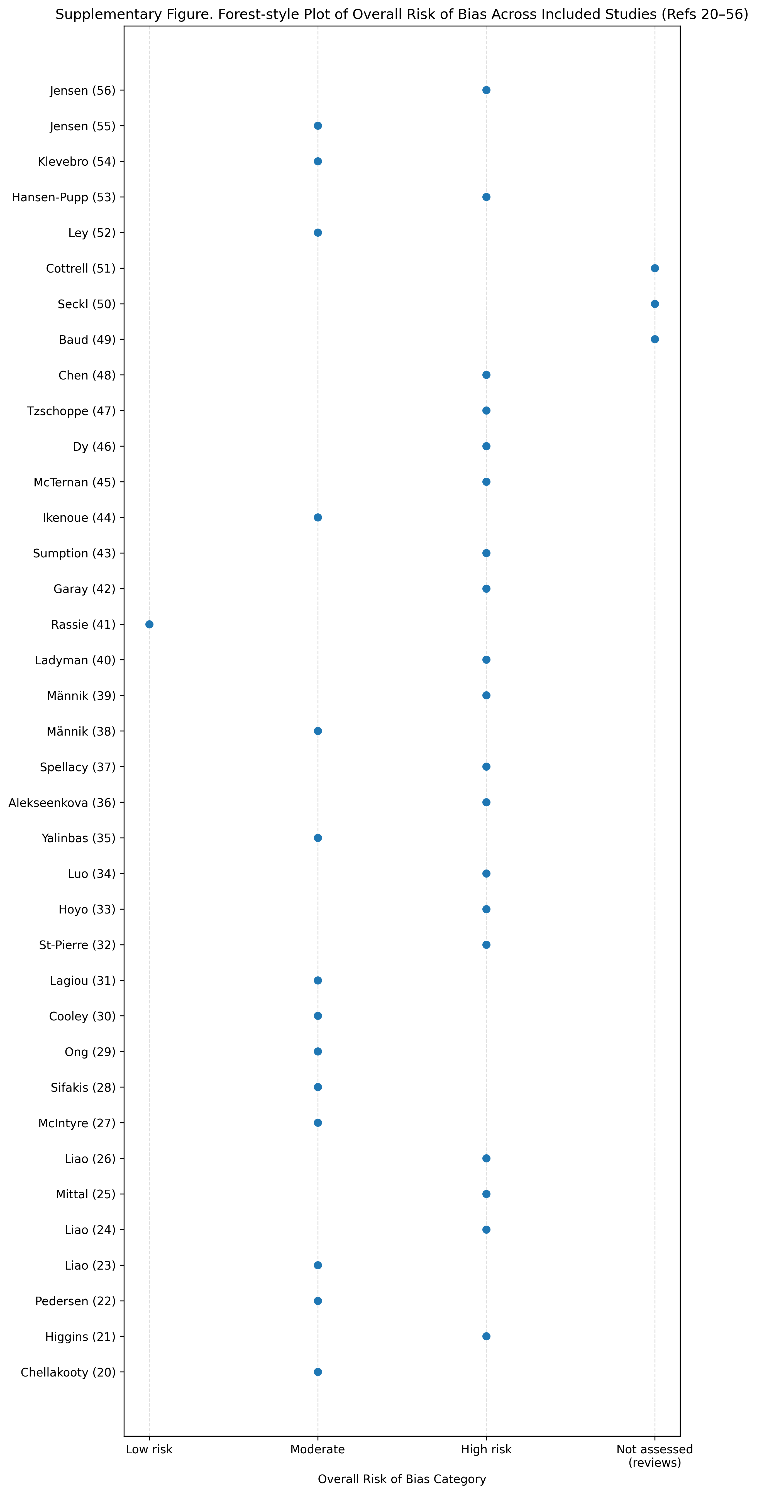


Forest-style summary of overall risk of bias for studies included in the review [20–56]. Each point represents an individual study, classified using Cochrane RoB 2 or ROBINS-I principles.
